# Genome-wide CRISPR screen identifies *LGALS2* as an oxidative stress-responsive gene with an inhibitory function on colon tumor growth

**DOI:** 10.1038/s41388-020-01523-5

**Published:** 2020-10-27

**Authors:** Haiwen Li, Lixia Zhao, Yeh Siang Lau, Chen Zhang, Renzhi Han

**Affiliations:** 1 Department of Surgery, Davis Heart and Lung Research Institute, Biomedical Sciences Graduate Program, Biophysics Graduate Program, The Ohio State University Wexner Medical Center, Columbus, OH 43210 USA; 2 Present Address: School of Healthcare and Medical Sciences, Sunway University, 47500 Bandar Sunway, Selangor, Malaysia

**Keywords:** High-throughput screening, Stress signalling

## Abstract

Colorectal cancer is the third leading cause of cancer-related deaths in the United States and the third most common cancer in men and women. Around 20% colon cancer cases are closely linked with colitis. Both environmental and genetic factors are thought to contribute to colon inflammation and tumor development. However, the genetic factors regulating colitis and colon tumorigenesis remain elusive. Since reactive oxygen species (ROS) is vitally involved in tissue inflammation and tumorigenesis, here we employed a genome-wide CRISPR knockout screening approach to systemically identify the genetic factors involved in the regulation of oxidative stress. Next generation sequencing (NGS) showed that over 600 gRNAs including the ones targeting *LGALS2* were highly enriched in cells survived after sublethal H_2_O_2_ challenge. *LGALS2* encodes the glycan-binding protein Galectin 2 (Gal2), which is predominantly expressed in the gastrointestinal tract and downregulated in human colon tumors. To examine the role of Gal2 in colitis, we employed the dextran sodium sulfate (DSS)-induced acute colitis model in mice with (WT) or without *Lgals2* (Gal2-KO) and showed that Gal2 deficiency ameliorated DSS-induced colitis. We further demonstrated that Gal2-KO mice developed significantly larger tumors than WT mice using Azoxymethane (AOM)/dextran sodium sulfate (DSS)-induced colorectal cancer model. We found that STAT3 phosphorylation was significantly increased in Gal2-deficient tumors as compared to those in WT mice. Gal2 overexpression decreased the proliferation of human colon tumor epithelial cells and blunted H_2_O_2_-induced STAT3 phosphorylation. Overall, our results demonstrate that Gal2 plays a suppressive role in colon tumor growth and highlights the therapeutic potential of Gal2 in colon cancer.

## Introduction

Colitis-associated cancer (CAC), one of the leading cause of cancer-related death, is a type of colon cancer, which is preceded by inflammatory bowel disease (IBD) [1, 2]. IBD including ulcerative colitis (UC) and Crohn’s disease (CD) is characterized by chronic relapsing and recurring inflammation of the gastrointestinal (GI) tract [3]. Dysfunction of the intestinal epithelial cell barrier and defective innate immune responses can lead to IBD and enhance the intestinal susceptibility to microbial invasion. This inflammatory niche as well as other secondary signals such as oxidative stress exerts many downstream effects like genomic mutation accumulation or oncogene activation, which can cause colon tumor development [4]. However, the genetic factors that regulate oxidative stress-mediated initiation and progression of colon cancer remain elusive.

Because of the rapid growth of tumor cells and infiltration of immune cells, cytokine release and oxidative stress play important roles in the initiation and progression of colon cancer. Mitochondria and membrane-bound NADPH oxidases are two main source of endogenous ROS. ROS can induce cell injury, increase epithelial barrier permeability, and promote luminal pathogen invasion, which in turn exacerbate inflammatory cell infiltration and contribute to tumorigenesis [5]. While excessive ROS production can cause cell death by activating intrinsic mitochondrial apoptotic signaling pathway, mildly elevated ROS could lead to sustained cell proliferation through many pathways such as STAT3 (signal transducer and activator of transcription 3) and MAPK (mitogen-activated protein kinase) [6, 7].

Beta-galactoside binding protein Galectin family has been implicated in ROS-mediated pathogenesis of IBD and CAC [8, 9]. Among these members, Gal2, highly expressed in GI tract and monocytes, was shown to play an important role in mucosal immune system [10, 11]. Genome-wide association studies found that Gal2 downregulation is linked to gastric and colorectal tumorigenesis [12–14]. Decreased Gal2 appeared to promote gastric cancer metastasis while increased serum Gal2 was found in colorectal cancer metastasis [12, 15]. However, the lack of studies with genetic animal models of Gal2 limits the capacity to uncover the role of endogenous Gal2 in inflammation and tumorigenesis.

In this study, we employed a genome-wide CRISPR screening approach to systemically identify genes involved in cell survival during oxidative stress. Over 600 guide RNAs (gRNAs) including the ones targeting *LGALS2* were highly enriched in survived cells after sublethal H_2_O_2_ challenge. DSS-induced colitis model and AOM/DSS-induced colorectal cancer model showed Galectin2 loss ameliorated experimental acute colitis but promoted tumor growth. Mechanically, Gal2 deficiency activated STAT3, promoting colorectal tumor cell growth. Overall, this study supports a suppressive role of Gal2 in the colorectal tumor development.

## Results

### Genome-wide CRISPR screening identified an oxidative stress responsive network

**Fig. 1:**
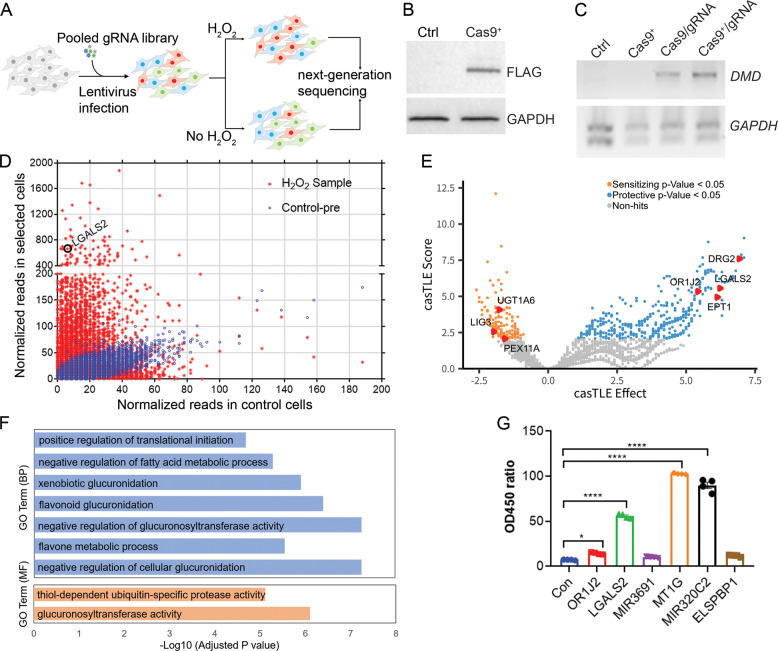
Genome-wide CRISPR screen identified oxidative stress-related genetic factors. **A** Diagram of genome-wide CRISPR screen. **B** Western blotting of Cas9 in stable Cas9-expressing HEK293 cells. **C** Genomic DNA PCR with the primers flanking the two gRNA target sites in the *DMD* gene detected a small deletion product in Cas9^+^ HEK293 cells transfected with the gRNA pair and in control HEK293 cells transfected with both Cas9 and gRNA pair, but not in control or Cas9^+^ cells without gRNA transfection. The scatter (**D**) and volcano (**E**) plots showing enrichment and depletion of specific gRNAs after H_2_O_2_ treatment. Control-pre cells are the Cas9^+^ HEK293 cells harboring the gRNA library before H_2_O_2_ treatment, while the control cells showing in the X-axis are the Cas9^+^ HEK293 cells harboring the gRNA library without H_2_O_2_, but otherwise similarly cultured as for the cells treated with H_2_O_2_. H_2_O_2_ sample represents Cas9^+^ HEK293 cells harboring the gRNA library with H_2_O_2_. **F** Gene ontology (GO) enrichment analysis of the genes with *p* < 0.05. Number of genes belonging to each category is indicated. BP biological process, MF molecular function. **G** Validation of the top six hits using the CCK8 viability assay. The OD450 ratio means the ratio of the OD450 value after H_2_O_2_ treatment for 6 days to the initial OD450 value on day 0. *p < 0.05, ****p < 0.0001 (one-way ANOVA).

To identify the genes associated with oxidative stress-induced cell death/survival, we performed genome-wide CRISPR knockout screening in HEK293 cells challenged with a sublethal dosage of H_2_O_2_ (Fig. 1A). We first established the HEK293 cells stably expressing Cas9, which was validated by Western blotting (Fig. 1B), and verified genome editing activity of these cells by transfecting them with a pair of single gRNAs targeting *DMD* to delete a large genomic region. As shown in Fig. 1C, transfection of the two gRNAs into the stable Cas9^+^ cell line resulted in the detection of a small polymerase chain reaction (PCR) product of 623 bp as predicted (Lane 4), similar to the control cells co-transfected with both Cas9 and gRNAs (Lane 3), indicating that the stable Cas9 confers efficient genome editing. No PCR product was efficiently amplified from mock-transfected control or Cas9^+^ HEK293 cells due to the large size of the region (~6.8 kb).

We established the dosage response of HEK293 to H_2_O_2_ treatment and the IC_50_ at 48 h was found to be ~0.8 mM (Supplementary Fig. S1A). We chose 0.5 mM for our screening assay, which resulted in 67% and 33% cell viability at 48 and 96 h treatment, respectively (Supplementary Fig. S1B). The Cas9-expressing HEK293 cells (~1 × 10^8^) were infected at a MOI of 0.5 with the lentivirus carrying the GeCKO v2 gRNA library A, containing 6.5 × 10^4^ gRNAs targeting over 20,000 genes [16], resulting in a library of HEK293 cells with each gRNA represented in more than 1000 cells. The gRNA-expressing cell library was enriched by puromycin selection for 7 days. Subsequently, the HEK293 cell library was treated with or without 0.5 mM H_2_O_2_ for 6 days. Genomic DNA was extracted from the surviving cells, and the gRNA sequences were amplified. The gRNA sequences in the selected cells were analyzed by next-generation sequencing (NGS). The data were analyzed by casTLE [17, 18]. A total of 625 gRNAs were found to be significantly enriched and 345 gRNAs depleted in HEK293 cells after H_2_O_2_ treatment as compared to the cells without H_2_O_2_ treatment (Supplementary Table S1). Interestingly, several of these targets were previously identified in HeLa cells with H_2_O_2_ treatment [18], such as EPT1, UGT1A6, LIG3, and PEX11A (Fig. 1D, E). However, we also found many new targets (Fig. 1D and Supplementary Table S1), likely due to different treatment conditions or cell types used between our study and the previous study. Gene ontology analysis found that the gRNAs for the glucuronosyltransferase genes (*UGT1A10*, *UGT1A9*, *UGT1A8*, *UGT1A6*, *UGT1A3*, *UGT1A1*, *UGT1A7*, *UGT1A4*, *UGT1A2*, *UGT1A5*) related to the flavone metabolism and the genes (*USP17*, *USP18*, *USP30*) associated with de-ubiquitinases were highly depleted in cells with H_2_O_2_ treatment (Fig. 1F and Supplementary Table S1). Interestingly, these genes have previously been linked to cellular protection and defense against oxidative stress [19].

Besides the known genes associated with response to oxidative stress, we are particularly interested in novel genes involved in regulating oxidative stress. We thus selected the top hits from our screening data including the one targeting *LGALS2* for further verification. To this end, we prepared lentiviral particles carrying several top gRNA sequences and individually transduced the Cas9-expressing HEK293 cells. The puromycin-selected Cas9/gRNA-expressing HEK293 cells were then treated with 0.5 mM H_2_O_2_ for 6 days. The viability of the cells was measured by Cell Counting Kit (CCK8) assay. In the presence of 0.5 mM H_2_O_2_, four of the six tested gRNAs (including OR1J2, *LGALS2*, *MT1G*, and *mir320C2*) showed highly significant effects on promoting cell growth (Fig. 1G). Taken together, our genome-wide CRISPR screening identified a ROS-responsive gene network in HEK293 cells.

### Gal2 was highly expressed in the mouse GI tract

**Fig. 2:**
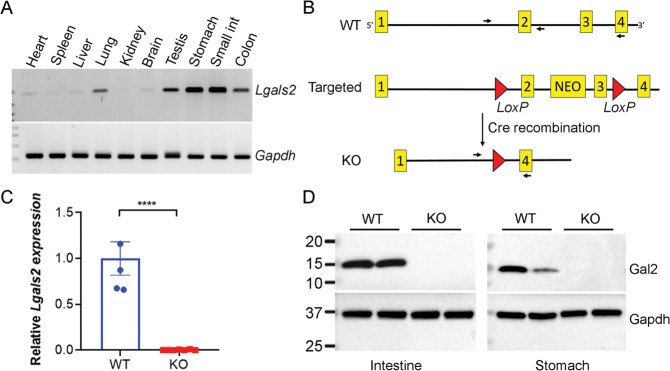
*Lgals2* was highly expressed in the gastrointestinal tract. **A** Expression of *Lgals2* in different mouse tissues examined by RT-PCR. **B** The diagram showing the strategy to generate Gal2-KO mice. **C** The expression of *Lgals2* in the intestines was examined by quantitative RT-PCR. **D** Western blotting showing the expression of Gal2 in the intestine and stomach of WT and Gal2-KO mice. ****p < 0.0001 (*t*-test).

Our attention was drawn toward *LGALS2* because several previous studies showed that Gal2 responded to H_2_O_2_ [20–22]. We analyzed the expression of Gal2 in mouse tissues and found that Gal2 was highly expressed in the GI tract (Fig. 2A). To study the physiological consequence of *Lgals2* gene disruption, we obtained a Gal2 knockout mouse line, in which the exons 2 and 3 of *Lgals2* were deleted (Fig. 2B). The Gal2-KO mouse was viable and fertile, and showed no obvious gross abnormality. As compared to wild-type (WT) mouse, the Gal2-KO mice showed disrupted Gal2 expression at both the transcript (Fig. 2C) and protein levels (Fig. 2D), while the expression of Gal2 in the heterozygous mouse was similar to WT mouse (Supplementary Fig. 2). In addition to Gal2, many of the other galectin family members were found to be expressed in mouse colon [8]. We found that the expression of these other galectin family members was not significantly altered in the colon of Gal2-KO mice (Supplementary Fig. 3).

### Disrupted Gal2 expression attenuated the development of acute colitis

**Fig. 3:**
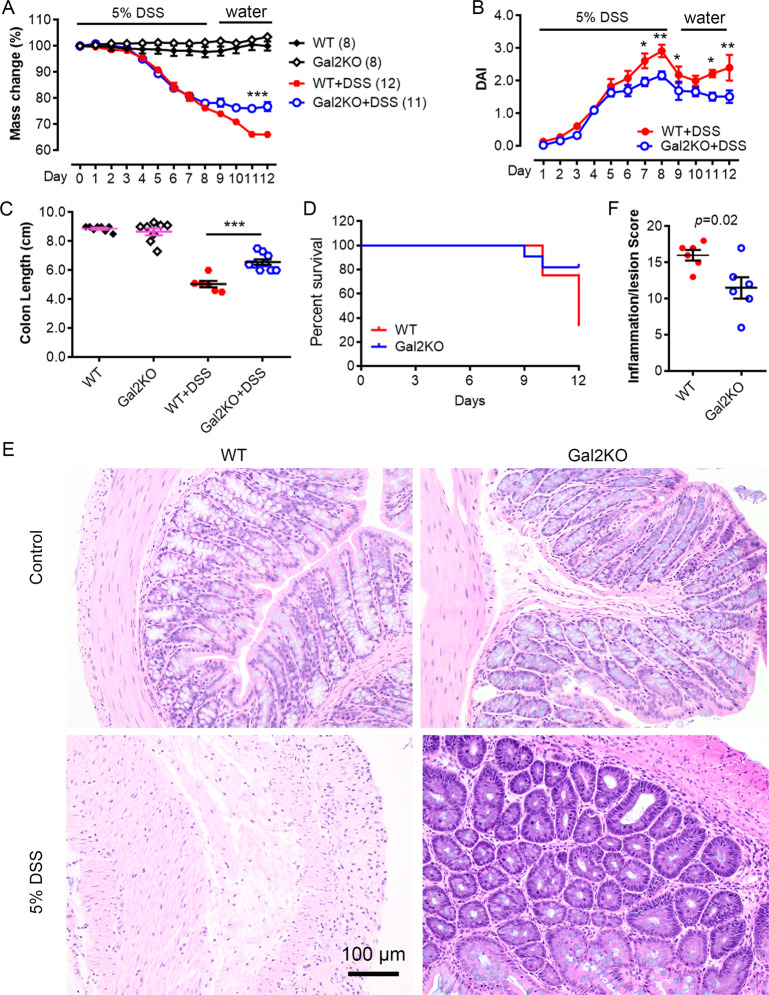
Gal2 deficiency attenuated DSS-induced acute colitis. **A** The body mass change over the 8-day course of DSS (5%) treatment and 4-day recovery period (regular drinking water). WT and Gal2-KO mice without DSS treatment served as controls. **B** The DAI of the WT and Gal2-KO mice over the course of DSS treatment. **C** The colon lengths of WT and Gal2-KO mice after DSS treatment. **D** Kaplan–Meier survival curve of age-matched WT and Gal2-KO mice following treatment with DSS. **E** Representative H&E-stained colon sections from WT and Gal2-KO mice with or without DSS treatment. Scale bar, 100 μm. **F** The inflammation/lesion score was blindly assessed by a board-certified pathologist. *p < 0.05, **p < 0.01, ***p < 0.001 (*t*-test for **A, B, F**; one-way ANOVA for **C**).

To investigate the role of Gal2 in the development of acute colitis, we treated WT and Gal2-KO mice with 5% DSS in drinking water for 8 days. Both groups started to lose body weight 3 days after the initiation of the treatment regime, but the Gal2-KO mice exhibited significantly less loss of body weight and displayed less death rate compared to WT controls (Fig. 3A, C). The Disease Activity Index (DAI) in the Gal2-KO mice was lower than the WT mice in the second half of the treatment regime and during the recovery phase (Fig. 3B). More than 60% WT mice died by day 12, while the Gal2-KO mice showed only 20% death (Fig. 3C). The colon length was significantly shortened in WT mice as compared to Gal2-KO mice (Fig. 3D). Histological examination of the colon samples by hematoxylin and eosin (H&E) staining showed that WT mice displayed extensive erosion, crypt damage, and infiltration of inflammatory cells into the colonic mucosa compared with Gal2-KO counterparts after DSS treatment (Fig. 3E and Supplementary Fig. 4). The overall histology score was significantly lower in Gal2-KO mice than that in WT mice (Fig. 3F). Taken together, these data suggest that disruption of Gal2 offers a protection against DSS-induced colitis in vivo.

### Gal2 depletion exacerbated the AOM/DSS-induced colorectal cancer

**Fig. 4:**
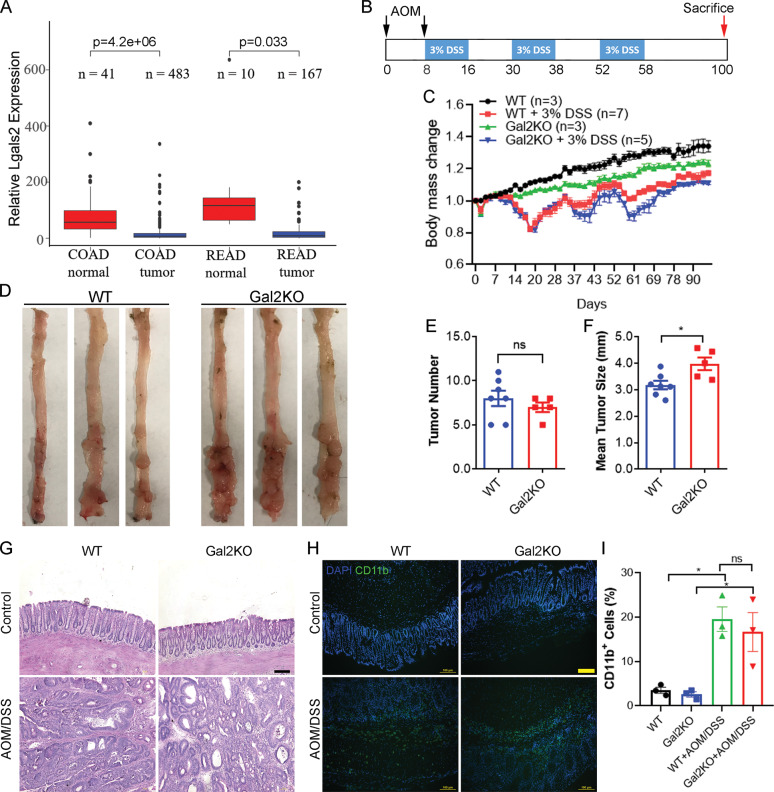
Gal2 disruption promoted the colon tumor growth. **A** Relative *LGALS2* transcript expression in normal human colons and colon tumors of patients (data obtained from GTEx and TCGA consortium, respectively). COAD colon adenocarcinoma, READ rectum adenocarcinoma. **B** Experimental scheme used to induce colon cancer in WT and Gal2-KO mice. AOM was given by i.p. injection, and DSS (3%) was given in drinking water. A consecutive 7-day DSS followed by 2 weeks of regular drinking water is considered as one cycle and the mice were treated for three cycles. **C** Changes in body mass of the mice over the course of the study. **D** The representative images of colon tumors from WT and Gal2-KO mice. **E** Number of tumors in WT and Gal2-KO mice induced by AOM and DSS treatment. **F** Mean tumor size in WT and Gal2-KO mice treated with AOM and DSS. **G** H&E staining of control colon and tumor tissues of WT and Gal2-KO mice. Scale bar: 100 µm. **H** CD11b and DAPI immunofluorescence staining images of colon tissues from WT and Gal2-KO mice with or without tumor induction. Scale bar: 100 µm. **I** Quantification of CD11b^+^ cells in colon tissues from WT and Gal2-KO mice with or without tumor induction. ns not statistically significant; *p < 0.05 (*t*-test for **E** and **F**; one-way ANOVA for **I**).

Next, we searched the TCGA database [23] and found that the expression of Gal2 was significantly decreased in colon tumor patients compared with normal persons, suggesting that Gal2 deficiency may be linked to tumorigenesis (Fig. 4A). Since combined administration of AOM and DSS has been widely used for establishing CAC [24], we exploited this model to study the role of Gal2 in the colorectal cancer. WT and Gal2-KO mice received intraperitoneal (i.p.) injections of 10 mg/kg AOM twice (one week apart), followed by three rounds of oral administration of 3% DSS in drinking water (Fig. 4B). The DSS treatment led to significant body weight loss in both WT and KO mice, but there was no significant difference between these two groups (Fig. 4C). At 6 weeks after the last treatment, all mice from both WT and KO groups developed detectable colorectal tumors, which were mainly distributed in the distal and middle sections regardless of their genotypes (Fig. 4D). The colon length showed no significant changes between the two groups (Supplementary Fig. 5). Although the number of tumors developed in each group was similar (Fig. 4E), the tumor size was significantly larger in Gal2-KO mice than that in WT mice (Fig. 4D, F). H&E staining showed disruption of normal epithelial layers in the tumor induction groups (Fig. 4G). These data suggest that Gal2 plays a suppressive role in tumor growth.

To characterize tissue inflammation in the mice with or without tumor induction, we stained the colon and tumor tissues with macrophage marker CD11b. There were no remarkable macrophages in control colon tissues of WT or Gal2-KO mice (Fig. 4H). In tumor-bearing animals, we observed macrophage infiltration in the submucosa and muscular layers of colon regardless of genotype (Fig. 4H, I), suggesting that Gal2 has little impact on chronic inflammation in AOM/DSS-induced colorectal tumors.

**Fig. 5:**
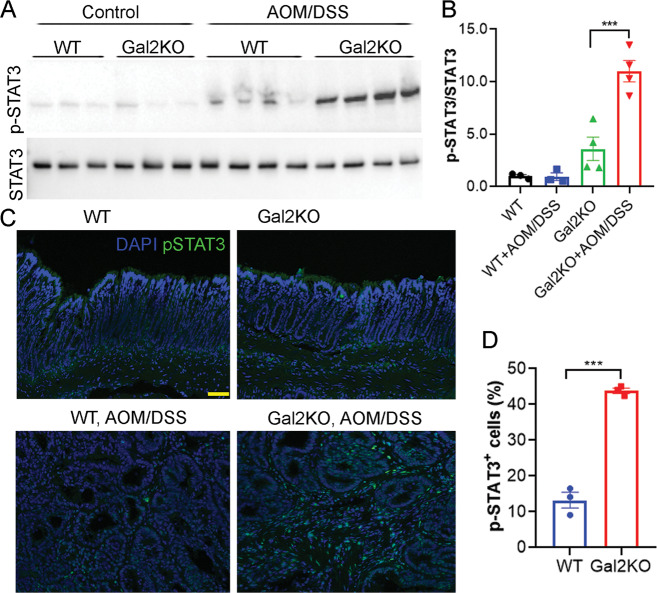
Gal2 disruption potentiated STAT3 activation in colorectal tumor. **A** Western blotting showing the expression of p-STAT3 and STAT3 in healthy colon or colon tumors. **B** Densitometry quantification of p-STAT3/total STAT3 for colons or colorectal tumors. ***p < 0.001 (one-way ANOVA). **C** Representative images of p-STAT3 immunostaining of colons or colon tumors. Scale bar: 50 μm. **D** Quantification of p-STAT3^+^ cells in colon tumors of WT and Gal2-KO mice. ***p < 0.001 (*t*-test).

During chronic inflammation, the inflammatory factors as well as oxidative stress activate STAT3, thereby resulting in the activation of cell survival and proliferation signalings [25, 26]. Western blotting showed that the phosphorylated-STAT3 (p-STAT3) was significantly increased in tumors as compared to healthy colons while Gal2 disruption showed a 2.5-fold further increase compared to WT tumors (Fig. 5A, B). Consistently, immunofluorescence staining also showed higher p-STAT3 level in the colon tumors of Gal2-KO mice than those of WT mice (Fig. 5C, D). These results suggest that increased STAT3 activation may contribute to faster tumor growth in Gal2-KO mice.

### Gal2 overexpression was susceptible to oxidative stress in HCT116

**Fig. 6:**
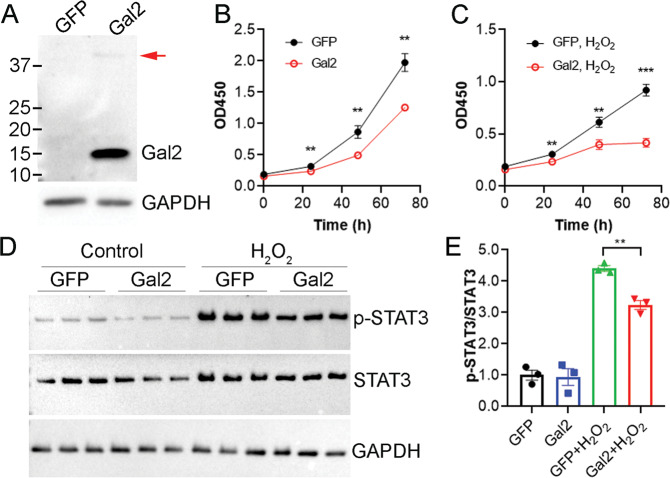
Gal2 overexpression inhibited HCT116 cell proliferation. **A** Western blotting showing the expression of Gal2 in stable Gal2-overexpressing HCT116 cells. The red arrow indicates the un-cleaved GFP-2A-Gal2 fusion protein. **B** The CCK8 proliferation assay of HCT116 with or without Gal2 overexpression. **C** The survival assay of HCT116 with or without Gal2 overexpression in the presence of 0.6 mM H_2_O_2_. **p < 0.01, ***p < 0.001 (*t*-test). **D** Western blotting showing the expression of p-STAT3 and STAT3 in HCT116 cells with or without Gal2 overexpression. **E** Densitometry quantification of p-STAT3/total STAT3 in HCT116 cells with or without Gal2 overexpression. **p < 0.01 (one-way ANOVA).

To further demonstrate the role of Gal2 in colorectal cancer cells under oxidative stress, we generated the HCT116 cells stably over-expressing Gal2 (Fig. 6A). Gal2 overexpression significantly decreased the proliferation of HCT116 cells (Fig. 6B), and increased their susceptibility to H_2_O_2_ (Fig. 6C). Similar results were observed in Caco-2 cancer cell line (Supplementary Fig. 6). Western blotting analysis showed that Gal2 overexpression did not significantly change the total STAT3 expression under the control condition (Fig. 6D, E). In response to H_2_O_2_ challenge, STAT3 phosphorylation was markedly increased, but Gal2 overexpression significantly blunted H_2_O_2_-induced STAT3 phosphorylation (Fig. 6D, E).

## Discussion

In this study, we applied a CRISPR knockout approach to reveal a genetic network involved in regulating cell survival in response to sublethal H_2_O_2_ challenge. Moreover, our data demonstrated that depletion of *Lgals2* rendered the cells resistant to H_2_O_2_ treatment. In vivo animal studies found that Gal2 depletion attenuated acute colitis and promoted colorectal tumor growth. In conclusion, Galectin2 played an important modulatory role in acute colitis and colon tumors.

It was previously shown by Paclik et al. [10] that exogenous Gal2 treatment reduced acute colitis by inducing T-cell apoptosis. Our present study showed that genetic disruption of Gal2 ameliorated acute colitis. This discrepancy may arise from several experimental differences between these two studies. First, the former study used exogenous Gal2 treatment while our study employed a loss-of-function approach to study the role of endogenous Gal2. Global administration of exogenous Gal2 may distinctly regulate the immune system and colon epithelial cells as compared to the local tissue-residing Gal2. Second, the former study used BALB/c mice while our study used mice with the genetic background C57BL/6J. Previous studies showed that different mouse strains exhibited different susceptibility to DSS [24, 27]. Consistent with this notion and our finding, Yildirim et al. found that Gal2 promoted artery inflammation by enhancing M1 macrophage skewing in C57BL/6J mice [10, 11].

Another interesting aspect of our finding is that Gal2 deficiency promoted the colon tumor development, although it ameliorated the acute colitis. Such paradoxical effects were previously reported in other cases such as IL-17F deficiency [28, 29], and may possibly be attributed to the common function of Gal2 in promoting cell death under oxidative stress in both epithelial cells and tumor cells. DSS mainly impairs the epithelial cells and destroys the mucosal barrier of colon, thereby leading to the penetration of microbiota into the tissues and eventually the inflammation. Endogenous Gal2 may inhibit the epithelial cell proliferation or promote their death under oxidative stress in acute colitis model, and thus disruption of Gal2 ameliorated the manifestation of acute colitis. On the other hand, Gal2 may also suppress tumor cell proliferation under oxidative stress as supported by our in vitro cell culture experiments and thus disruption of Gal2 accelerated tumor growth. It is worthy of note that the dose of DSS used for the tumor model was lower than that used for the acute colitis model. It is possible that under the lower dose of DSS, the impact of Gal2 disruption on epithelial cell death and thus the colitis development is less prominent.

Many members of the galectin family were reported to be involved in tumorigenesis with different effects. Califice et al. [30] demonstrated that overexpression of Gal3 in the cytoplasm of prostate cancer cells induced invasion, anchorage‑independent tumor growth, and reduced apoptosis, while nuclear overexpression resulted in the opposite biological activities. However, overexpression of Gal4 induced cell cycle arrest and reduced cell migration/mobility while sensitizing cells to camptothecin-induced apoptosis in colorectal cancer [31, 32]. Further studies showed that Gal4 induced downregulation of β-catenin, Dvl2, TCF1, TCF4, c-Myc, LRP6, and cyclin D1 expression levels while upregulating p21, p15 Naked 1, and Ephrin B1 [31, 33]. In addition, Gal8 has been shown to repress the colon tumor migration and Gal9 has been exhibited to reduce myelomas tumor growth by inhibiting JNK and p38 signaling [34, 35]. Our data reveal for the first time that Gal2 has a suppressive function on colon tumor growth likely via regulating the STAT3-mediated cell survival signaling.

Genome-wide CRISPR screening has been increasingly applied to study various signaling pathways in different disease conditions. For example, genome-wide CRISPR screening has been used to identify genes that are involved in many different biological processes such as myoblast fusion [36], tumor growth and metastasis [37, 38], and virus infection [39–41], demonstrating the power of genome-wide CRISPR screening to interrogate the genetic network in different biological systems. In addition to *LGALS2*, our study identified numerous other genes involved in regulating cellular responses to oxidative stress. The gRNAs targeting nearly 15 homologies of *USP17* and 10 members of the *UTG* family are depleted following H_2_O_2_ challenge, suggesting that disruption of these genes make the cells vulnerable to oxidative stress. These results are consistent with the previous studies showing that *USP17* regulates Redox signaling by stabilizing NAPDH oxidase [42] and the UTG genes play an essential role in the flavone metabolism, an essential metabolite for cell survival under oxidative stress [43, 44]. Our screening also identified many novel genes, whose disruption make the cells resistant to oxidative stress such as *MT1G* and microRNA *MIR320C2*. *MT1G*, a known tumor suppressor, was previously shown to regulate the cell viability under ROS [45]. During the preparation of this manuscript, a similar study also exploited the CRISPR Knockout library to identify the ROS-responsible genes in HeLa and K562 cells [18]. Taken together, these screening results represent a rich resource for the study of oxidative stress and highlight a number of potential targets that might be used for therapeutic intervention.

## Material and methods

### Mice

All animal studies were reviewed and approved by the Institutional Animal Care and Use Committee (IACUC) of the Ohio State University. Male C57BL/6 mice were purchased from The Jackson Laboratory (Bar Harbor, ME, USA). The Gal2-KO mice (B6(FVB)-Lgals2tm1.1Cfg/M-mucd) were obtained from Mutant Mouse Resource & Research Centers, UC Davis and maintained in our barrier facility. In Gal2-KO mice, the exons 2 and 3 were deleted via a Cre-mediated recombination by crossing with a Zp3-cre mice [46]. Identification of the mutant mice was performed by PCR genotyping of genomic DNA prepared from ear clips with the following primers. The KO allele was amplified with a forward primer located in the upstream of the first intron of Gal2 (5′-CAACGGGAAACTACAACATC-3′) and a reverse primer located in fourth exon of Gal2 (5′-GTGCTCAGATAGAGGAAACAAG-3′), and the WT allele was amplified using the same forward primer but a different reverse primer located in the second intron of Gal2 (5′-GCTAAGGTCTTCTGAGGTGC-3′). The WT and KO allele would produce a 666-bp and 655-bp band, respectively.

### DSS-induced colitis model

Acute colitis was induced in male C57BL/6J and Gal2-KO mice at the age of 10 weeks by administering 5% (wt/vol) DSS (DSS; MW 36,000–50,000; MP Biochemicals) in drinking water for 8 days. The groups were designed to detect differences in body mass and disease index between DSS-treated-wild-type (*n* = 12) versus Gal2-KO (*n* = 11) mice. A power calculation was not performed. Mice, randomly grouped to receive regular drinking water or DSS-containing water, were weighed every day and the percentage of body weight change of each mouse was calculated. Clinical scores are a combination of weight loss, rectal bleeding, and stool consistency [47]. Mice were sacrificed at day 10–12. Part of colons was fixed in 4% buffered paraformaldehyde and other sections of the colon were freshly snap-frozen and preserved in −80 °C for further analyses. Histopathology of colon samples was blindly assessed by a board-certified veterinary clinician at The OSU Comparative Pathology and Mouse Phenotyping Shared Resource, and scored as previously described [27, 48]. All mice were included in the analyses.

### AOM/DSS-induced colorectal cancer model

Male C57BL/6J and Gal2-KO mice at 10 weeks of age, randomly selected, were injected on day 0 with AOM (10 mg/kg; Sigma-Aldrich) by i.p. The groups were designed to detect differences in tumor burden between wild-type (*n* = 7) versus Gal2-KO (*n* = 5) mice. A power calculation was not performed. On day 7, mice were i.p. injected with AOM again (10 mg/kg; Sigma-Aldrich). On day 8, DSS was given in the drinking water (3% wt/vol) for 8 days (unless otherwise specified), followed by 14 days normal water. This DSS treatment regime was repeated for two more cycles. Mice were sacrificed and tissues were collected for analysis between day 90 and 100. The tumors were excised, washed, and counted. Colons were preserved either directly in liquid nitrogen for molecular/biochemical experiments or optimal cutting temperature (OCT)-embedded for cryosection. Investigators were blinded to group allocation during tumor growth assessment and analysis.

### RNA extraction and quantitative RT-PCR analysis

Total RNA was extracted from mouse colons with Trizol. First-strand cDNA was synthesized using RevertAid RT Reverse Transcription Kit (Life Technologies, Carlsbad, CA, USA). Real-time PCR was performed using PerfeCTa SYBR Green FastMix (QuantaBio, USA) in CFX384 Real-time PCR Detection Systems (Bio-Rad). Samples were normalized for expression of *Gapdh* and analyzed by the 2^−^^ΔΔCt^ method.

### Western blotting

The colon samples from WT and Gal2-KO mice were lysed with cold RIPA buffer supplemented with protease inhibitors, and the extracted proteins were quantified by DC^TM^ Protein Assay Reagent (BioRad). The extracted protein samples were separated by stain-free SDS-PAGE gels (BioRad, 4-15%) and transferred onto Nitrocellulose Membranes (0.45 μm). Primary antibodies including the goat polyclonal anti-Gal2 (STJ24400, 1:500, St John’s Laboratory Ltd, London, UK), anti-STAT3 (#9139, 1:1000), anti-p-STAT3 (#9145, 1:1000), and anti-GAPDH (MAB374, 1:2000) were purchased from Cell Signaling Technology (Danvers, MA). Secondary HRP-conjugated goat anti-mouse (1:4000), goat anti-rabbit (1:4000) antibodies were obtained from Cell Signaling Technology. The membranes were developed using ECL western blotting substrate (Pierce Biotechnology, Rockford, IL) and scanned by ChemiDoc XRS+ system (BioRad, Hercules, CA). Western blots were quantified using Image Lab 6.0.1 software (Bio-Rad Laboratories, Hercules, CA) according to the manufacturer’s instruction.

### Cell culture and ROS treatment

HEK293 cells were cultured in Dulbecco’s modified eagle’s medium (Sigma, St. Louis, MO, USA) containing 10% fetal bovine serum (FBS) and 1% penicillin–streptomycin (Invitrogen). The stable Cas9-expressing HEK293 cells were established by lentiviral transduction and Blasticidin S selection. The Cas9-expressing HEK293 cells were verified by transfection with a pair of gRNAs targeting the intron 52 (5′gATTAAGACTAACGAAAGCGA) and 53 (5′gAAGCTCTAGTCATATTCGTG) of human *DMD* allele to delete a ~6.2-kb genomic region. A pair of primers specific for intron 52 (5′AGGTCAAGGGTGAAAAAGCAT) and 53 (5′CCAGAGTCCTCTTGCCCTAGT) beyond the gRNA target sites was used to genotype the cells for genomic editing. Cells were transfected with the designated plasmids by polyethylenimine as previously described [49]. The cell viability was examined by the CCK-8 cell counting kit (Dojindo Molecular Technologies, MD, USA). HEK293 cells were prepared in 96-well plates (5 × 10^3^ cells/plate) with 0.5-mM H_2_O_2_ at the indicated incubation time. Absorbance was measured at 450 nm according to the manufacturer’s instructions. The pLVX-GFP and pLVX-EGFP-P2A-Gal2 lentiviral particles were packaged according to the previous study [50], and then infected HCT116 and Caco-2 cells. Transduced HCT116 or Caco-2 cells were selected with 1-μg/mL puromycin for 48 h. The cell lines (HEK293, HCT116, and Caco-2) were obtained from ATCC and were not authenticated or tested for mycoplasma contamination.

### CRISPR genome-wide screen

The GeCKO v2 library was obtained from Addgene (Watertown, MA). Library virus production was conducted as described in the instruction. Briefly, HEK293 cells were seeded per 15-cm dish, and the cells were transfected 24 h later with a mix of 8-μg lentiviral vector containing the library, 8-μg packaging vector psPAX2, 4-μg envelope vector pMD2.G, 60-μL X-tremeGENETM HP DNA transfection reagent (Sigma-Aldrich, St. Louis, MO), and 2.0 mL Opti-MEM medium (Life Technologies, Carlsbad, CA). The virus-containing medium was harvested 48–72 h after transfection, centrifuged at 1500 rpm for 5 min, and frozen at −80 °C. The HEK293 cells were infected with the GeCKO v2 library lentiviruses and selected with 2 µg/mL puromycin for 1 week. The selected HEK293 cells were expanded and the gRNA library was verified by NGS. An aliquot of HEK293 cell library (~6.5 × 10^7^ cells) was challenged without or with 0.5 mM H_2_O_2_ for 6 days and the survived cells were grew up for genomic DNA extraction using the DNeasy Blood & Tissue Kits (Qiagen, Germantown, MD) and resuspended in Buffer EB (10 mM Tris-HCl, pH 7.5). The gRNA sequences were amplified by two-step PCR as described previously [16]. Primers used to amplify gRNAs are listed in Supplementary Table S2. The resulting gRNA libraries were sequenced on an Illumina HiSeq 2500 system. Bioinformatical analysis of genome-wide screen was performed using casTLE according to the previous studies [17, 18], which is a maximum likelihood estimator that uses a background of negative control gRNAs as a null model to estimate gene effect and score. The identified gRNAs were listed in Supplementary Table S1.

### H&E and immunofluorescence staining

The large intestines were removed and embedded in OCT compound, snap-frozen using isopentane chilled in liquid nitrogen, and kept at −80 °C until use. Cryosections were prepared using a cryostat Leica CM3054. For H&E staining, transversely oriented sections (10 μm) were cut at midpoint and stained as previously described [51]. The samples were digitally imaged using a Nikon Ti-E inverted fluorescence microscope equipped with a Lumenera Infinity Color CCD camera, and a Nikon Super Fluor 20 × 0.75 NA objective lens (Nikon Inc., Melville, NY, USA). The digital images were processed using NIS-Elements AR version 4.30 (Nikon, Melville, NY).

Frozen tissue sections (10 µm) were fixed with 4% paraformaldehyde for 15 min at room temperature. After washing with PBS, the slides were blocked with 3% BSA for 1 h. The slides were incubated with primary antibodies against p-STAT3 (#9145, 1:1000, CST) at 4 °C overnight. After that, the slides were washed extensively with PBS and incubated with Alexa Fluor 488 (donkey anti-rabbit IgG, Invitrogen) for 1 h at room temperature. The slides were sealed with VECTASHIELD Antifade Mounting Medium with DAPI (Vector Laboratory, Burlingame, CA). All images were taken with a Nikon Ti-E fluorescence microscope (magnification ×20) (Nikon, Melville, NY).

### Bioinformatic analysis

Gene expression profiles of 51 normal colon samples and 648 colon tumor samples were derived from four datasets (TCGA CC, GSE14333, GSE8671, and GSE41258). The downloaded clinical data were matched to the mRNA expression profile. Data analysis was performed as previously described [52].

### Statistical analysis

All in vitro experimental data were repeated three times, and all in vivo studies were repeated twice. Data are expressed as mean ± the standard error of the mean. The data were first tested for normality using the Shapiro–Wilk test, and found to have similar variance. Statistical differences were determined by two-tailed unpaired Student’s *t* test for two groups and one-way ANOVA with Turkey’s post tests for multiple group comparisons using Prism 8 (Graphpad Software, La Jolla, California). A *p* value < 0.05 was considered to be significant.

## Supplementary information

Supplementary Information

Supplemental Table S1

Supplemental Table S2

## Data Availability

The accession number for the raw sequencing files for all screens performed in this paper is SRA: PRJNA663915. The authors declare that all data supporting the findings of this study are available in the article and its Supplementary Information files are available from the corresponding author on reasonable request.
