# Supplementary material for: Genome-wide CRISPR screen identifies LGALS2 as an oxidative stress-responsive gene with an inhibitory function on colon tumor growth
Source: Oncogene. 2020 Oct 27;40(1):177–88. doi: 10.1038/s41388-020-01523-5 (PMC7790754; doi:10.1038/s41388-020-01523-5)
Supplement: Supplementary file 1 — Supplementary Information [file 41388_2020_1523_MOESM1_ESM.docx]

**Supplementary Information**

**Supplemental Table S1.** The oxidative stress-responsible genes identified by the genome-wide CRISPR screen and Gene Orthologs analysis of these candidates (p<0.05).

**Supplemental Table S2.** List of primers used in the study.


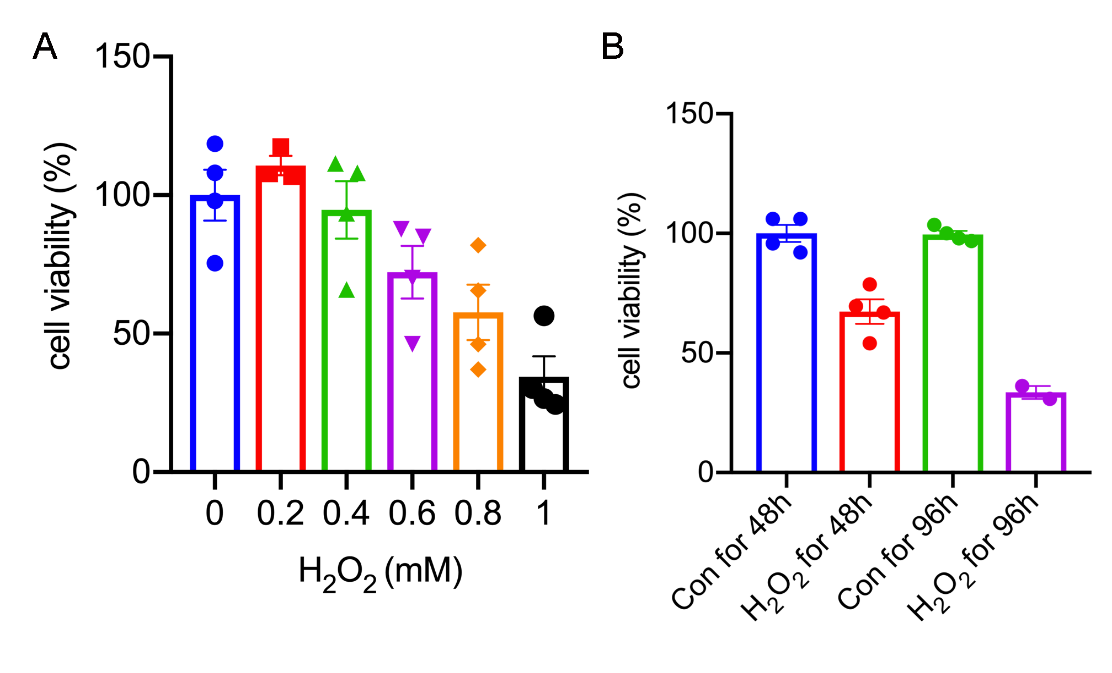


**Supplemental Figure 1.** Establishment of the dose-response curve of H_2_O_2_ in HEK293 cell survival. (A) H_2_O_2_ dose-dependently inhibited HEK293 cell survival. (B) HEK293 cell viability at 48 and 96 hours following treatment with 0.5 mM H_2_O_2_.


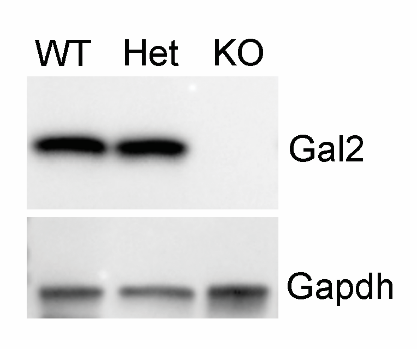


**Supplemental Figure 2.** Western blot analysis of Gal2 in the colons of WT, heterozygous (Het) and homozygous (KO) Gal2-KO mice.


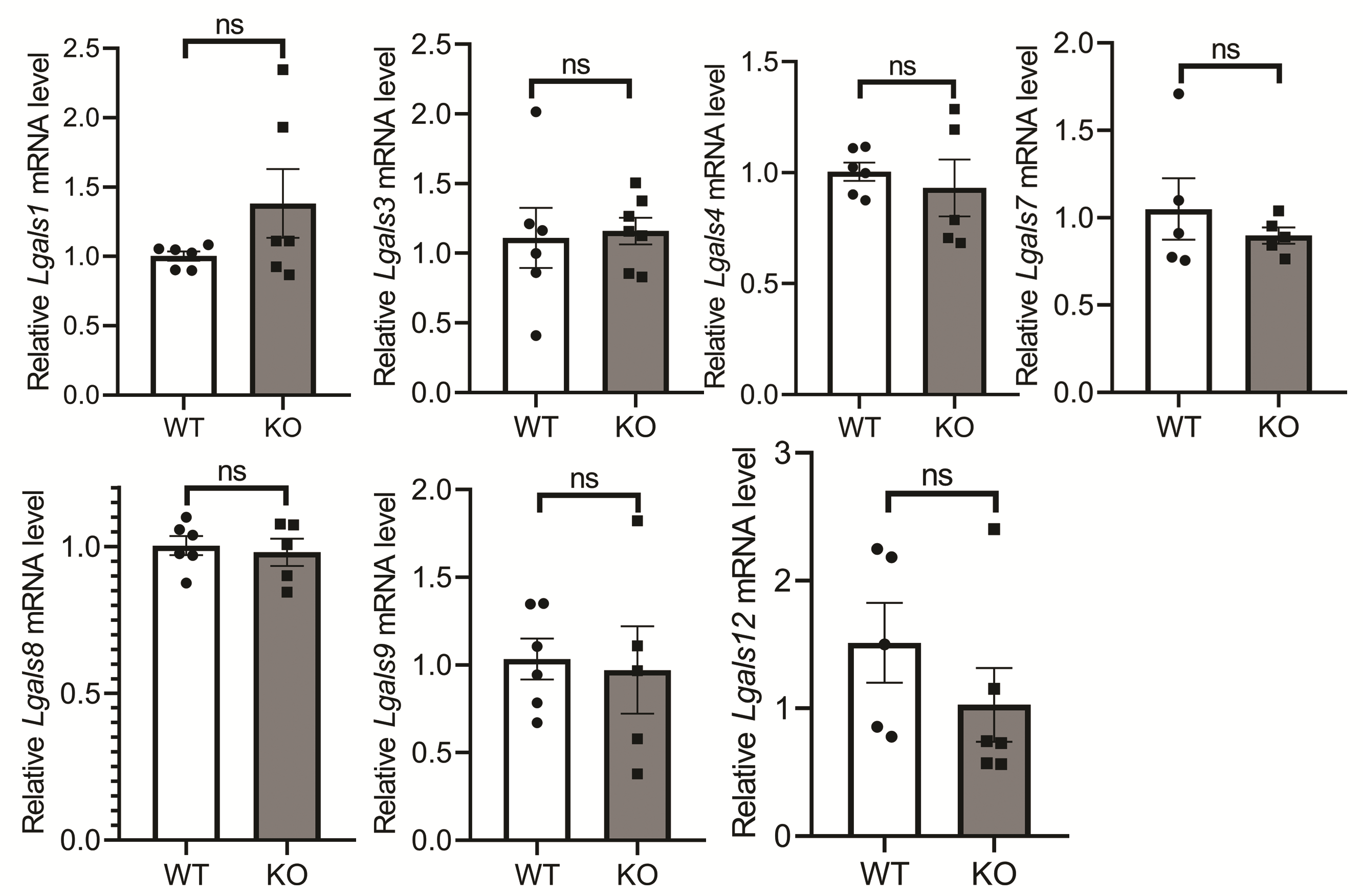


**Supplemental Figure 3.** Expression of galectin family genes in the colons of WT and Gal2-KO mice examined by quantitative RT-PCR.


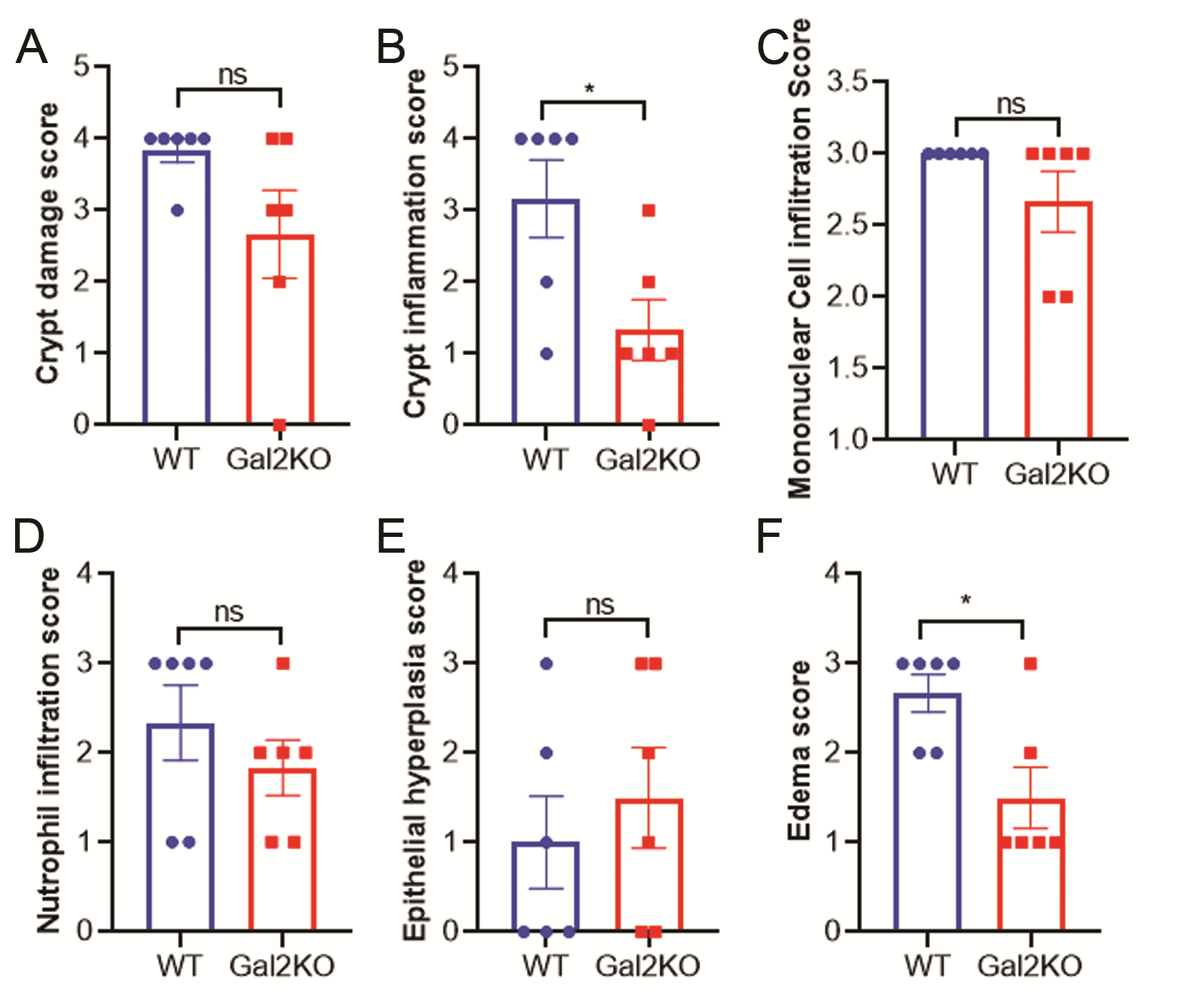


**Supplemental Figure 4.** Histological scores of colon samples in DSS-treated WT and Gal2-KO mice. Crypt damage (A), crypt inflammation (B), mononuclear cell infiltration (C), neutrophil infiltration (D), epithelial hyperplasia (E), and edema (F) were scored on H&E-stained colon samples from WT and Gal2-KO mice treated with DSS.


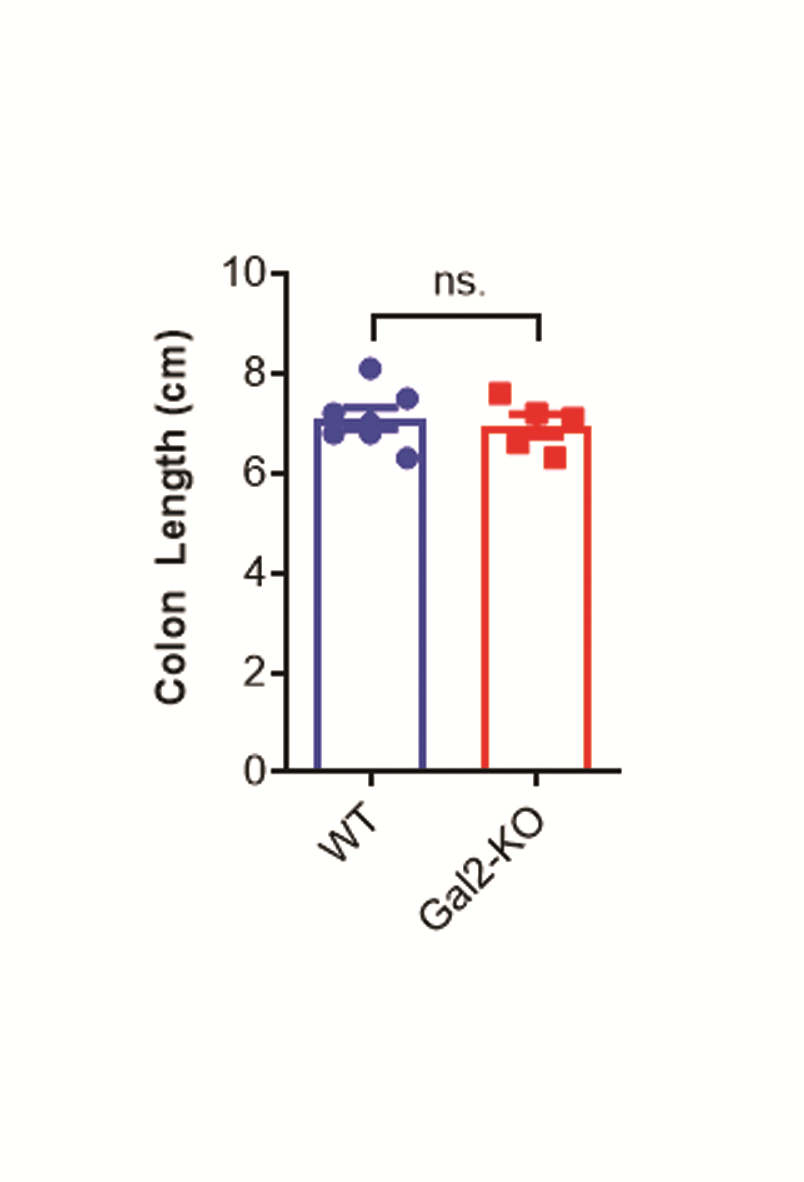


**Supplemental Figure 5.** The colon length of WT and Gal2-KO mice treated with AOM and DSS.


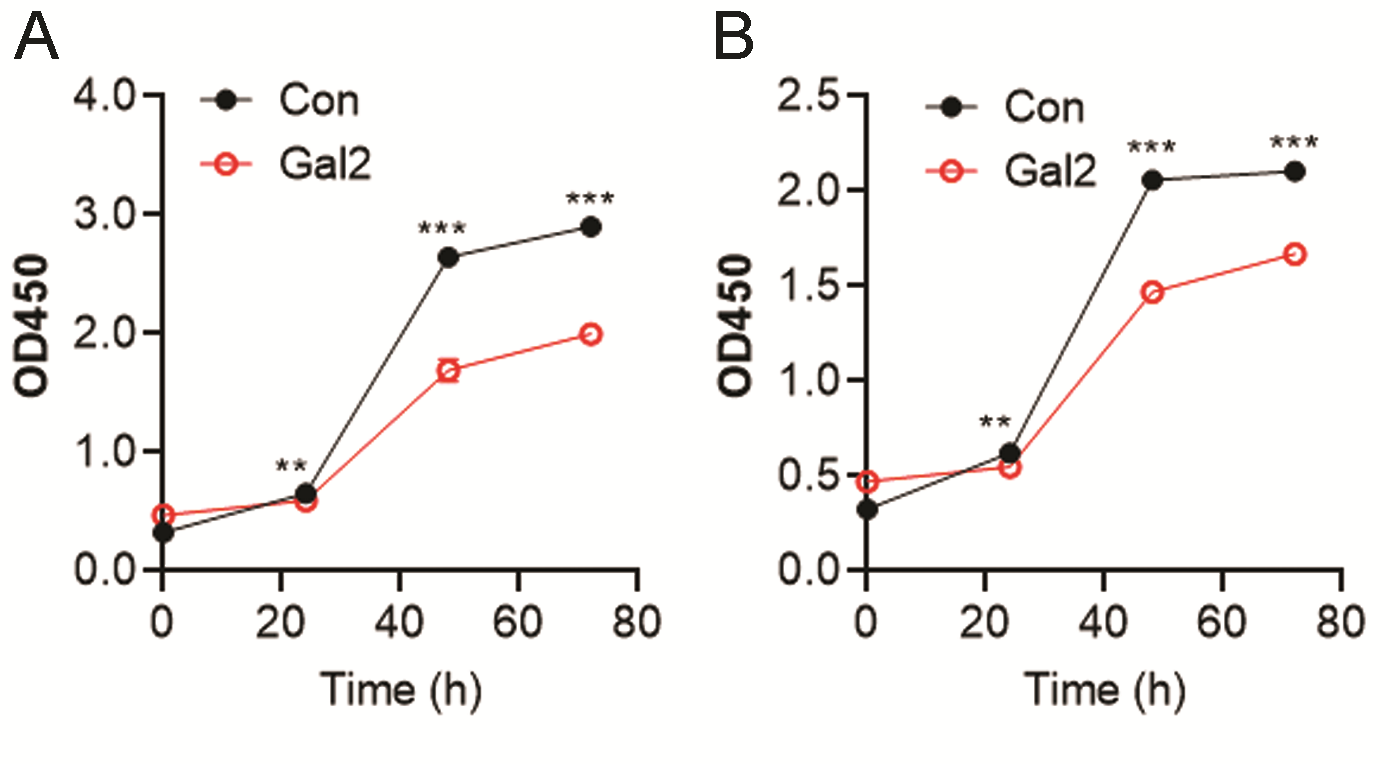


**Supplemental Figure 6.** Effect of Gal2 overexpression on Caco-2 cell proliferation. (A) The CCK8 proliferation assay of Caco-2 cells with or without Gal2 overexpression. (B) The survival assay of Caco-2 cells with or without Gal2 overexpression in the presence of 0.6 mM H_2_O_2_. **p<0.01, ***p<0.001 (*t*-test).
